# Does Unilateral Lumbosacral Radiculopathy Affect the Association between Lumbar Spinal Muscle Morphometry and Bone Mineral Density?

**DOI:** 10.3390/ijerph182413155

**Published:** 2021-12-14

**Authors:** Minjung Kim, Jinmann Chon, Seung Ah Lee, Yunsoo Soh, Myung Chul Yoo, Yeocheon Yun, Seongmin Choi, Min Gyun Kim

**Affiliations:** 1 Department of Physical Medicine and Rehabilitation Medicine, Kyung Hee University Medical Center, Seoul 02447, Korea; koko9638@naver.com (M.K.); soyuns@gmail.com (Y.S.); famousir@naver.com (M.C.Y.); yunsn123@naver.com (Y.Y.); ysisminee@naver.com (S.C.); 2 Department of Physical Medicine and Rehabilitation, Kyung Hee University Hospital at Gangdong, Seoul 05278, Korea; lsarang80@gmail.com (S.A.L.); kimmgdg@naver.com (M.G.K.)

**Keywords:** bone mineral density, psoas, multifidus, erector spinae, cross-sectional area, radiculopathy, fatty degeneration

## Abstract

Age-related degenerative changes lead to a gradual decrease in bone mineral density (BMD) and muscle mass. We aimed to assess the effects of decreased BMD and lumbar denervation on lumbar spinal muscle morphometry and the relationship between BMD and lumbar spinal muscular morphometry, respectively. Eighty-one patients, aged 50–85 years, diagnosed with unilateral lumbosacral radiculopathy based on electrodiagnostic studies between January 2016 and April 2021 were enrolled. BMD T scores in the lumbar spine and hip were measured using dual-energy X-ray absorptiometry. The cross-sectional area (CSA) of the psoas, multifidus, and erector spinae located in the middle of the lumbar spine, between the L3 and L4 and between the L4 and L5 levels, respectively, was measured using axial MRI. Functional CSA (FCSA) was defined as the CSA of lean muscle mass. Pearson correlation analyses were performed to evaluate the association between BMD T scores and the CSA, FCSA, and the ratio of the FCSA to the CSA (functional ratio) for each side. The CSA of lumbar spinal muscles showed no significant correlation with lumbar BMD. The FCSA and functional ratio of lumbar spinal muscles were significantly correlated with lumbar BMD. There was no correlation between femur BMD and lumbar spinal muscle morphometry.

## 1. Introduction

Osteoporosis is defined as a bone mineral density (BMD) that is 2.5 standard deviations below the peak mean bone mass in young healthy adults. It is a skeletal disease characterized by low bone mass and microarchitectural deterioration of bone tissue, with a consequent increase in bone fragility and fracture susceptibility. There are multiple factors affecting bone density, including nutrition, environment, hormone, genetic factors, and the interactions between these factors [1]. Furthermore, aging is an important risk factor for osteoporosis [2]. The prevalence of osteoporosis has not yet been adequately documented in several parts of the world. The prevalence of osteoporosis in Korean women over the age of 50 is 37.3% and the prevalence of osteoporotic fractures gradually increased from 2008 to 2016 [3]. The risk of developing osteoporosis and fractures increases with age [4]. This has emerged as a major problem affecting the quality of life and increasing the socioeconomic burdens of the elderly.

Muscle mass also decreases with increasing age, resulting in decreased physical activity and an increased risk of falls and fractures [5]. Paraspinal musculature is also influenced by physiological processes, such as aging [6]. In addition, few studies have demonstrated changes in the multifidus and erector spinae muscles in patients with lumbar radiculopathy [7,8,9]. Thus, similar to BMD, changes in muscle composition are affected by multiple factors.

Importantly, a decline in muscle mass is not the only factor that contributes to the deterioration of muscle function in the context of muscle aging. Other factors underpinning muscle quality include muscle composition, aerobic capacity and metabolism, fatty infiltration of muscle, insulin resistance, fibrosis, and neural activation [10].

The physiological or pathological mechanisms underlying the interactions between muscles and bones are unclear. It has been suggested that sarcopenia and osteoporosis may co-exist, and share a common etiology. Furthermore, lean muscle mass and BMD are influenced by common genetic factors. Previous studies suggest that the vitamin D receptor (VDR) gene may be one of the common genetic factors. The same polymorphism of the VDR gene (Fok I) has been found to be associated with BMD and lean mass [11,12,13,14].

Various methods have been used to assess muscle mass in patients with sarcopenia. Muscle mass is commonly assessed using dual-energy X-ray absorptiometry or bioimpedance analysis, handgrip strength, and physical performance with the short physical performance battery or usual gait speed [15]. The measurement of the cross-sectional area (CSA) of the psoas muscle is also a reliable marker for sarcopenia [16,17].

There is evidence of a mechanistic interrelationship between muscle and bone in sarcopenic individuals who are at a high risk of osteoporosis [4]. In particular, the lumbar muscles support and maintain stability of the lumbar spine [5,11]. The body’s muscle mass can be largely distinguished by fat mass and lean mass. Numerous studies have revealed that fat mass and lean mass are correlated with whole body BMD, but studies comparing the correlation between BMD and muscle mass or muscle quality are limited. Among the lumbar spinal muscles, multifidus and erector spinae are innervated by the dorsal ramus medial branch of the segmental nerve. The psoas muscle is innervated by the lumbar nerve root and lumbar plexus. In this study, we assessed whether decreased BMD was related to lumbar spinal muscle morphometry. If there was a relationship between BMD and lumbar spinal muscle morphometry, we assumed that denervation of the lumbar nerve root, which innervates the paraspinal muscle, could affect this relationship. Therefore, we also evaluated whether lumbosacral nerve root denervation affected the relationship between BMD and the quality of the lumbar spinal muscles.

## 2. Materials and Methods

### 2.1. Subjects

This retrospective study included patients who were diagnosed with unilateral lumbosacral polyradiculopathy based on electrodiagnostic studies at the clinic of the Department of Rehabilitation Medicine of Kyung Hee University Hospital between January 2016 and April 2021.

The inclusion criteria were as follows: (1) female sex, (2) age between 50 and 85 years, (3) clinical symptoms of unilateral radiculopathy, and (4) the diagnosis of unilateral lumbosacral polyradiculopathy based on an electrodiagnostic study. The electrodiagnostic criteria for polyradiculopathy were as follows: (1) abnormal spontaneous activity in unilateral paraspinal muscles and/or (2) abnormal spontaneous activity or abnormal motor unit morphology consistent with nerve injury (polyphasic, large amplitude, increased duration) or decreased recruitment patterns in the lower extremity muscles innervated by the same dermatome but by different peripheral nerves.

The exclusion criteria were as follows: (1) absence of lumbar magnetic resonance imaging (MRI) findings, (2) absence of BMD measurements, (3) previous spinal surgery, (4) spinal tumor, (5) spinal fractures, and (6) spinal infections such as vertebral osteomyelitis or discitis.

A total of 263 patients underwent electrodiagnostic studies during the study period; among them, 182 were excluded. Finally, 81 subjects were enrolled in this study. This study was approved by the Institutional Review Board of Kyung Hee University Hospital (IRB number: 2021-08-055).

### 2.2. Measures and Procedures

All MRI (3.0T, Siemens, Magnetom Vida, Germany) examinations of the lumbar spine were performed. BMD was measured using DISCOVERY-W fan-beam densitometer with dual X-ray absorptiometry (DEXA, GE Lunar Prodigy, Lunar Corporation, Madison, WI, USA). BMD T scores in the lumbar spine were measured as an average of L1 through L4 levels, except for the level with degenerative changes in the vertebral column that restrict data interpretation. BMD T scores in the hip were measured at the femoral neck and whole femur.

All patients underwent electrodiagnostic studies and DEXA within three months before or after MRI examinations. Axial T2-weighted MRI signals at the L3/4 and L4/5 intervertebral discs were measured between the bottom margin of the upper vertebra and the top margin of the lower vertebra. The CSA of the lumbar spinal muscles, including the multifidus, erector spinae, and psoas, was calculated by drawing a region of interest (ROI) around each muscle using the PiView program (Infinitt, Seoul, Korea). Axial magnetic resonance (MR) images were exported from a picture archiving and communication system, and image reconstruction was performed using an image processing software (Image J, version 1.53e; National Institute of Health, Wayne Rasband, DC, USA).

**Figure 1:**
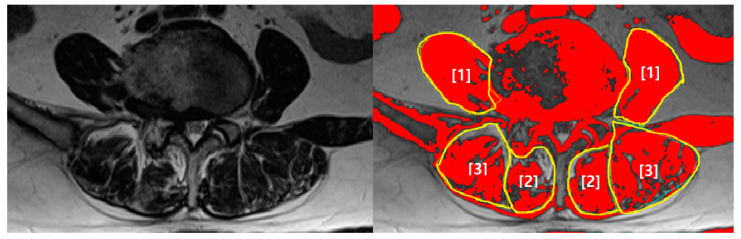
Measurement of CSAs and FCSAs on a representative T2-weighted magnetic resonance (MR) image. (**Left**): original T2-weighted MR image. (**Right**): T2-weighted MR image showing CSAs as the area surrounded by the yellow lines and FCSAs as the red colored areas. [1]: Psoas, [2]: Multifidus, [3]: Erector spinae; CSA, cross-sectional area; FCSA, functional cross-sectional area.

The CSAs of the lumbar spinal muscles were measured separately on the right and left sides. The side of radiculopathy was defined as the involved side, and the side that was not affected by radiculopathy was defined as the uninvolved side. CSA was measured by drawing lines along the fascia around the multifidus, erector spinae, and psoas muscles. Functional CSA (FCSA) was defined as the CSA of the lean muscle mass, excluding muscular fatty degeneration. The low MR signal intensity of the muscle was differentiated from the high MR signal intensity of fat using Image J [18]. CSA and FCSA were calculated by defining the threshold between the signal intensity of the muscle and fat [19]. The measurement of FCSA included lean muscle mass alone, except for fatty infiltration above a certain MR signal intensity threshold. The threshold for each patient was defined as the highest MR signal intensity in grayscale obtained by selecting ROIs in the muscle portion alone and excluding fat tissue. Functional ratio was defined as the ratio of FCSA to CSA and was calculated as the percentage of lean muscle area, including fatty degeneration of the lumbar spinal muscles (Figure 1).

### 2.3. Statistical Analysis

Statistical analyses were performed using the Statistical Package for Social Sciences (version 25.0; SPSS Inc., Chicago, IL, USA). The relationship between BMD and the quality or quantity of the paraspinal muscles was analyzed using Pearson correlation analysis. Generalized linear mixed models were used to evaluate the effect of lumbosacral radiculopathy on the relationship between lumbar BMD and lumbar spinal muscle morphometry, including FCSA and functional ratio. Statistical significance was set at *p*-value less than 0.05.

## 3. Results

### 3.1. Subject Demographics and Characteristics

This study included 81 female patients aged between 50 and 85 years (mean 65.4 ± 7.8 years). All patients had lumbar disc herniation, including disc bulging, protrusion, extrusion, and sequestration, on MR images. Thirty-eight female patients had spinal stenosis, and five patients had spondylolisthesis at the L4 or L5 level. Thirty-four patients were diagnosed with osteoporosis based on BMD (Table 1). Based on electromyography findings, 37 and 44 patients were diagnosed with right-sided and left-sided radiculopathy, respectively (Table 1).

### 3.2. Relationship between BMD and Lumbar Spinal Muscle Morphometry

Pearson correlation analysis showed no correlation between the CSA, which was calculated by combining the area of muscle and fat in patients with and without radiculopathy, and BMD (Table 2). However, the FCSAs of the psoas and multifidus muscles on both sides at the L3/4 and L4/5 levels showed a statistically significant correlation with lumbar BMD (*p* < 0.05). There was no significant correlation between the FCSA of the erector spinae and lumbar BMD (Table 3). The functional ratios of the psoas, multifidus, and erector spinae on both sides at the L3/4 and L4/5 levels also showed a statistically significant correlation with lumbar BMD (*p* < 0.05) (Table 4). However, femur neck BMD and total femur BMD were not statistically correlated with the CSA, FCSA, or the functional ratio of the lumbar spinal muscles (Table 2, Table 3 and Table 4).

The sum of the FCSA and functional ratio of the psoas and multifidus on both the involved and uninvolved sides showed a significant association with lumbar BMD (*p* < 0.05). There was no significant association between the CSA and lumbar BMD. The CSA and FCSA of the erector spinae were not associated with lumbar BMD, but the functional ratio of the erector spinae was statistically associated with lumbar BMD at the L3/4 and L4/5 levels (Table 5).

We observed no significant differences in the FCSA and functional ratio of lumbar spinal muscles between the involved and uninvolved sides (Table 6).

## 4. Discussion

A few studies have explored the relationship between BMD and the age-related decline in lean mass or sarcopenia; however, their results were inconsistent [20]. In a study of 14,429 Koreans, low muscle mass was significantly associated with osteoporosis in both men and women in all age groups, except for men aged 50–64 years [21]. A meta-analysis of 44 studies showed that while both lean muscle mass and fat mass were associated with BMD, lean muscle mass was a more important determinant of BMD than fat mass in men and women of all ages and ethnicities [22]. However, a study by Walsh et al. observed no significant correlation between BMD and skeletal muscle index in 213 healthy women after adjusting for physical activity [23].

In this study, FCSA, defined as the CSA of lean muscle mass of the lumbar spinal muscle, and lumbar BMD were related. In particular, the lean muscle mass of the psoas and multifidus muscles was related to lumbar BMD. The relationship between the lean muscle mass of the erector spinae and lumbar BMD was weak. This study also showed a statistically significant relationship between the fat infiltration of lumbar spinal muscle and lumbar BMD. The CSA of lumbar spinal muscle, which included both fat and muscle area, showed little association with lumbar BMD. These findings were consistent with those of a previous study; according to the study, the replacement of muscle with fat may not significantly alter the CSA of the muscle although fat infiltration is a sign of muscle atrophy [24].

This study showed no association between femur BMD and the CSA, FCSA, and fatty infiltration of lumbar spinal muscles. This indicates that although the morphometry of adjacent muscles and BMD are related, lumbar spinal muscle morphometry itself does not significantly affect femur BMD.

The factors affecting the causal relationship between bones and muscles are yet to be fully identified. A variety of factors can affect the relationship between muscle quality and BMD, including vitamin D deficiency, testosterone, estrogen, and insulin growth factors, none of which are independent variables [25,26].

Recent studies that adjusted for the gravity effect of weight-bearing showed a negative relationship between body fat and bone mass [27]. Mechanical loading influences BMD distribution; it can also influence both lean body mass and body fat content due to gravity [22].

In addition, various studies have shown that paraspinal muscles affect the balance of the lumbar spine, and are involved in various diseases, indicating a correlation between the paraspinal muscles and spinal BMD [28,29]. This correlation was evaluated in the present study. One study showed a significant correlation between the fatty degeneration of paraspinal muscles and the progression of spinal compression fractures, but the CSA of the paraspinal muscles did not affect spinal collapse, such as vertebral compression fracture. This result is consistent with our findings, suggesting that lean muscle mass is more important for vertebral stability than total muscle mass [30].

Muscles are powerful osteogenesis stimulators, and the estimated torque generated by the muscles can result in a difference in bone structure. Therefore, surrounding muscles have the capability to change adjacent skeletal structure. This ability can explain why lumbar spinal muscle is particularly related to the adjacent lumbar BMD, rather than femur BMD. In addition, since lean muscle alone is a more important factor for forming muscle power than lean and fat mass combined, paraspinal lean mass and fat infiltration were more related to the lumbar BMD as previously noted [31,32]. A reduction in paraspinal muscle strength is associated with osteoporosis. Back extensor muscle weakness compresses the vertebrae in fragile osteoporotic spines. Improvement in back muscle strength is required to support the vertebrae. Therefore, rehabilitation treatments such as back muscle strengthening exercises can be effective in improving lumbar BMD. As muscle strength is proportional to the CSA of the muscle, the CSA of paraspinal muscle can be used to evaluate lumbar muscular strength [33,34]. 

In this study, the association between femur BMD and lumbar spinal muscle morphometry was not statistically significant. Therefore, we did not study this relationship in the presence or absence of radiculopathy. This is the first study to reveal that the presence or absence of lumbosacral polyradiculopathy does not affect the association between lumbar BMD and lumbar spinal muscle morphometry. Our findings suggest that the reduction in bone density is associated with lumbar muscle morphometry but not with lumbar nerve root denervation.

This study has some limitations. First, the number of subjects who underwent all three examinations, DEXA, lumbar MRI, and electromyography tests and were diagnosed with lumbosacral radiculopathy was small. Second, this was a retrospective study; further prospective studies are required to assess other factors, such as dietary habits and daily activities. Third, unilateral polyradiculopathy in some patients included in this study did not fully damage nerve function or lead to denervation. Fourth, the examinations such as MRI, BMD, and electrodiagnostic study were performed by different experts in different departments, which is another limitation of this study. Fifth, the subjects of this study were limited to postmenopausal women of varying ages. Further studies should subdivide the age group.

## 5. Conclusions

Lumbar BMD T scores were correlated with the functional muscle mass and fatty degeneration of the lumbar spinal muscles, particular in the psoas and multifidus muscles. This indicates that the size and quality of the lumbar muscles are important factors affecting the lumbar BMD. However, femur BMD showed no correlation with lumbar spinal muscle morphometry. The presence or absence of radiculopathy had no significant effect on the relationship between lumbar BMD and the quality of lumbar muscles.

## Figures and Tables

**Table 1:** Patients’ baseline characteristics.

Variables	
Number of patients	81
Age (years)	65.4 ± 7.8
Height (cm)	154.8 ± 5.1
Weight (kg)	59.3 ± 9.4
Body mass index (kg/m^2^)	24.8 ± 3.9
BMD at the lumbar spine (g/cm^2^)	−1.706 ± 1.113
BMD at the femur neck (g/cm^2^)	−1.619 ± 1.065
Disc herniation (n)	81
Spinal stenosis (n)	38
Spondylolisthesis at the L4 or L5 level	5
Osteoporosis (n)	34
Right-sided radiculopathy (n)	37
Left-sided radiculopathy (n)	44

BMD, bone mineral density. Data are expressed as mean ± standard deviation.

**Table 2:** Pearson correlation analysis to analyze the correlation between the CSA of lumbar spinal muscles and lumbar BMD.

		Psoas		Multifidus		Erector Spinae	
		Involved Side	Uninvolved Side	Involved Side	Uninvolved Side	Involved Side	Uninvolved Side
Lumbar BMD							
L3/4	Coefficient	0.082	0.112	0.028	−0.021	0.009	−0.038
	*p*-value	0.469	0.321	0.804	0.853	0.933	0.733
L4/5	Coefficient	0.260	0.181	0.062	−0.011	−0.089	−0.068
	*p*-value	0.019 *	0.106	0.584	0.924	0.431	0.549
Femur neck BMD							
L3/4	Coefficient	0.014	0.167	−0.071	−0.082	0.152	0.103
	*p*-value	0.904	0.153	0.545	0.484	0.192	0.381
L4/5	Coefficient	0.075	0.142	0.110	0.087	−0.038	−0.039
	*p*-value	0.524	0.225	0.349	0.458	0.748	0.737
Total femur BMD							
L3/4	Coefficient	0.071	0.135	−0.057	−0.076	0.248	0.177
	*p*-value	0.542	0.224	0.624	0.516	0.031 *	0.126
L4/5	Coefficient	0.080	0.113	0.096	0.028	0.061	0.008
	*p*-value	0.492	0.333	0.408	0.810	0.601	0.945

CSA, cross-sectional area; BMD, bone mineral density. * *p* < 0.05.

**Table 3:** Pearson correlation analysis to analyze the correlation between the FCSA of lumbar spinal muscles and lumbar BMD.

		Psoas		Multifidus		Erector Spinae	
		Involved Side	Uninvolved Side	Involved Side	Uninvolved Side	Involved Side	Uninvolved Side
Lumbar BMD							
L3/4	Coefficient	0.264	0.300	0.326	0.293	0.177	0.198
	*p*-value	0.017 *	0.007 *	0.003 *	0.008 *	0.114	0.076
L4/5	Coefficient	0.334	0.340	0.309	0.287	0.128	0.226
	*p*-value	0.002 *	0.002 *	0.005 *	0.009 *	0.255	0.043 *
Femur neck BMD							
L3/4	Coefficient	0.083	0.250	0.056	−0.047	−0.001	0.044
	*p*-value	0.479	0.030 *	0.636	0.691	0.996	0.710
L4/5	Coefficient	0.110	0.147	−0.054	−0.130	0.009	0.056
	*p*-value	0.349	0.207	0.643	0.266	0.940	0.635
Total femur BMD							
L3/4	Coefficient	0.126	0.219	0.178	0.055	0.200	0.222
	*p*-value	0.279	0.057	0.125	0.639	0.084	0.053
L4/5	Coefficient	0.133	0.144	0.059	−0.059	0.156	0.209
	*p*-value	0.250	0.215	0.611	0.615	0.178	0.070

FCSA, functional cross-sectional area; BMD, bone mineral density. * *p* < 0.05.

**Table 4:** Pearson correlation analysis to analyze the correlation between the functional ratio of the lumbar spinal muscles and lumbar BMD.

		Psoas		Multifidus		Erector Spinae	
		Involved Side	Uninvolved Side	Involved Side	Uninvolved Side	Involved Side	Uninvolved Side
Lumbar BMD							
L3/4	Coefficient	0.225	0.359	0.360	0.388	0.227	0.303
	*p*-value	0.043 *	0.001 *	0.001 *	<0.001 *	0.041 *	0.006 *
L4/5	Coefficient	0.264	0.356	0.321	0.359	0.226	0.324
	*p*-value	0.017 *	0.001 *	0.003 *	0.001 *	0.042 *	0.003 *
Femur neck BMD							
L3/4	Coefficient	0.133	0.235	0.073	−0.034	−0.060	0.004
	*p*-value	0.257	0.042	0.536	0.773	0.609	0.972
L4/5	Coefficient	0.104	0.114	−0.118	−0.178	−0.009	0.094
	*p*-value	0.373	0.329	0.312	0.127	0.938	0.423
Total femur BMD							
L3/4	Coefficient	0.086	0.221	0.237	0.111	0.100	0.170
	*p*-value	0.459	0.056	0.039	0.338	0.391	0.141
L4/5	Coefficient	0.132	0.131	0.031	−0.047	0.135	0.232
	*p*-value	0.257	0.261	0.791	0.684	0.245	0.044 *

BMD, bone mineral density. * *p* < 0.05.

**Table 5:** Pearson correlation analysis to analyze the correlation between lumbar spinal muscular morphometry and lumbar BMD.

		Psoas			Multifidus			Erector Spinae		
		CSA	FCSA	Functional Ratio	CSA	FCSA	Functional Ratio	CSA	FCSA	Functional Ratio
Lumbar BMD										
L3/4	Coefficient	0.102	0.334	0.283	0.003	0.325	0.398	−0.015	0.200	0.281
	*p*-value	0.363	0.002 *	0.010 *	0.980	0.003 *	<0.001 *	0.894	0.074	0.011 *
L4/5	Coefficient	0.236	0.358	0.344	0.026	0.309	0.356	−0.085	0.186	0.295
	*p*-value	0.034 *	0.001 *	0.002 *	0.817	0.005 *	0.001 *	0.449	0.096	0.007 *

CSA, cross-sectional area; FCSA, functional cross-sectional area; BMD, bone mineral density. * *p* < 0.05.

**Table 6:** Generalized linear mixed models to evaluate the effect of lumbosacral radiculopathy on the relationship between lumbar BMD and lumbar spinal muscle morphometry on the involved and uninvolved sides.

		Psoas		Multifidus		Erector Spinae	
		FCSA	Functional Ratio	FCSA	Functional Ratio	FCSA	Functional Ratio
Lumbar BMD							
L3/4	Coefficient	−0.001	−0.024	0.000	−0.003	0.000	−0.009
	*p*-value	0.142	0.004 *	0.960	0.784	0.626	0.487
L4/5	Coefficient	0.000	−0.005	0.000	−0.003	−0.001	−0.008
	*p*-value	0.963	0.657	0.857	0.738	0.478	0.478

FCSA, functional cross-sectional area; BMD, bone mineral density. * *p* < 0.05.

## Data Availability

The data presented in this study are available from the corresponding author on request. The data are not publicly available due to privacy matters.
